# Targeting the DNA Damage Response in Cancer

**DOI:** 10.1002/mco2.71020

**Published:** 2026-09-23

**Authors:** Yihuan Qiao, Boyu Kang, Jian Zhang, Rui Zhang, Jipeng Li

**Affiliations:** ^1^ Department of Digestive Surgery Xijing Hospital of Digestive Diseases Fourth Military Medical University Xi'an China; ^2^ State Key Laboratory of Holistic Integrative Management of Gastrointestinal Cancers and National Clinical Research Center for Digestive Diseases Xijing Hospital of Digestive Diseases Fourth Military Medical University Xi'an China; ^3^ State Key Laboratory of Holistic Integrative Management of Gastrointestinal Cancers and Department of Biochemistry and Molecular Biology Fourth Military Medical University Xi'an Shaanxi China; ^4^ Department of Experiment Surgery Xijing Hospital of Fourth Military Medical University Xi'an China

**Keywords:** biomarkers, cGAS–STING, DNA damage response, immune checkpoint blockade, PARP inhibitor, tumor immune microenvironment

## Abstract

The DNA damage response (DDR) is increasingly recognized not only as a genome‐maintenance network and source of synthetic‐lethal vulnerabilities, but also as a regulator of tumor immunity. DDR defects and pharmacologic inhibition can increase neoantigen formation, generate micronuclei and cytosolic nucleic acids, activate cGAS–STING, DNA‐PK–NF‐κB, and ERV‐driven RIG‐I/MDA5–MAVS signaling, and alter antigen presentation and immune checkpoint expression. However, the same processes may also promote chronic interferon tolerance, PD‐L1 induction, lymphocyte stress, myeloid suppression, extracellular cGAMP degradation, stromal exclusion, and treatment resistance. This review frames DDR targeting around damage‐to‐immunity conversion, asking when DNA damage becomes a productive antitumor immune signal and when it does not. We summarize targetable DDR modules across cancer types, examine tumor‐intrinsic and host‐cell sensing mechanisms, and discuss antigen‐processing defects, STING regulation, and microenvironmental barriers that shape divergent outcomes. We further review PARP, ATR, WEE1, ATM, and DNA‐PK inhibitor combinations with immunotherapy and other treatment modalities, emphasizing clinical evidence, scheduling, and host‐cell toxicity. Finally, we propose a modular biomarker framework integrating genomic, functional, immune‐context, spatial and dynamic readouts to guide patient selection, treatment adaptation, and precision therapeutic decision‐making.

## Graphical abstract

Targeting the DNA damage response (DDR) reshapes both tumor vulnerability and antitumor immunity. DNA lesions and DDR inhibitors can promote cytosolic nucleic‐acid sensing, antigen presentation, and immune activation, but can also induce adaptive immune escape. Integrating DDR states, immune context, and dynamic biomarkers may enable rational combination therapy and precision patient stratification.

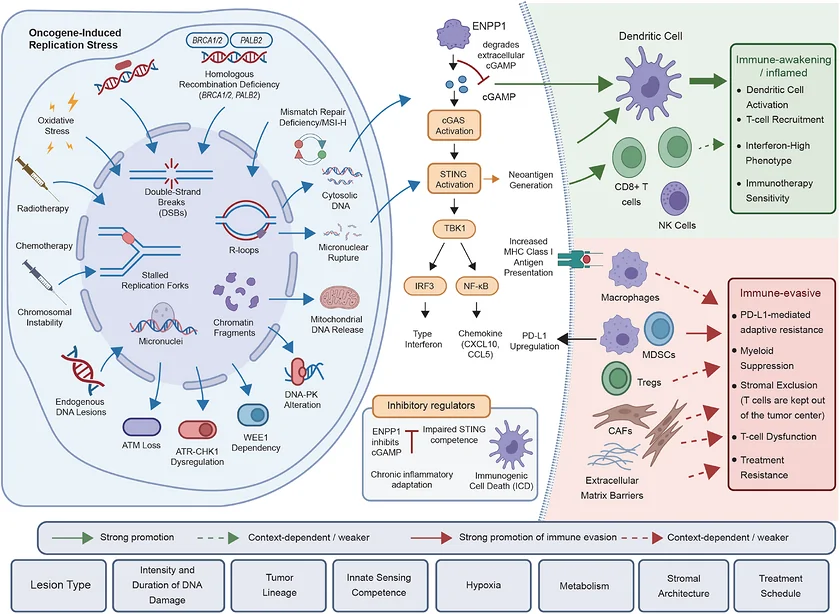

## 1 Introduction

The DNA damage response (DDR) was historically defined as a set of pathways that detects DNA lesions, arrests the cell cycle, and repairs the genome. This classical view remains essential: tumors depend on homologous recombination (HR), non‐homologous end joining (NHEJ), mismatch repair (MMR), base and nucleotide excision repair, Fanconi anemia signaling, PAR metabolism, and checkpoint kinases such as ATM, ATR, CHK1, WEE1, and DNA‐PK to survive oncogene‐driven replication stress and exogenous genotoxic therapy [1, 2, 3, 4, 5, 6, 7]. Recent work has also highlighted the immunomodulatory consequences of PARP and broader DDR targeting [8, 9, 10, 11]. Foundational BRCA–PARP synthetic‐lethality studies established the therapeutic principle [12, 13], which has since been translated clinically across ovarian, breast, prostate, and pancreatic cancers [14, 15, 16, 17, 18]. Other DDR targets, including ATR, WEE1, ATM, and DNA‐PK, are discussed in the sections below.

A second layer of DDR biology is now equally important for cancer therapy. Unrepaired or mis‐repaired DNA damage is not immunologically silent. DNA damage can engage inflammatory and innate‐sensing programs through multiple DDR‐linked mechanisms [19, 20, 21, 22, 23]. Canonical studies established cGAS/cGAMP sensing and micronuclear surveillance as central routes linking genomic instability to innate immunity [24, 25, 26, 27, 28]. Radiotherapy models further demonstrated STING‐dependent antitumor immunity and TREX1‐mediated control of cytosolic DNA [29, 30, 31]. However, genomic injury should not be equated with immune benefit. The same damage signal can induce PD‐L1, exhaust T cells, stress proliferating lymphocytes, recruit suppressive macrophages, amplify adenosine‐rich extracellular metabolism, or reinforce stromal exclusion [10, 32, 33, 34, 35, 36, 37, 38, 39]. Thus, the therapeutic question is not whether DDR perturbation causes damage, but whether that damage is converted into useful immunity in a particular tissue context.

What this review adds is a framework centered on damage‐to‐immunity conversion. Rather than summarizing DDR targets or immune combinations in isolation, we ask why the same DDR defect can generate immune activation in one lineage and immune suppression in another. We integrate canonical cGAS–STING sensing with DNA‐PK‐dependent inflammation, ERV‐driven RLR–MAVS viral mimicry, antigen‐processing machinery, host‐cell sensing, stromal and myeloid barriers, clinical trial evidence, pharmacologic scheduling, and biomarker‐guided stratification. The review proceeds from the targetable DDR landscape to immune mechanisms, then to therapeutic combinations and finally to an integrated biomarker model based on genomic, functional, immune‐context, spatial, and dynamic monitoring. This sequence is intended to help readers move from static genotype to a treatment‐relevant immune state.

## 2 The Targetable DDR Landscape in Cancer

DDR targeting now spans many cancers and cannot be reduced to one pathway or one drug class. This section first summarizes the major targetable DDR modules, then explains how their immune consequences are conditioned by tumor lineage, lesion duration, dose intensity, and stromal architecture. The goal is to build a wider title‐aligned foundation before focusing on tumor immunity and biomarker‐guided combinations.

### 2.1 DDR Pathways as Therapeutic Liabilities

Historically, each repair pathway was studied as a relatively separate biochemical solution to a particular lesion. In tumors, however, pathway boundaries are fluid. A stalled replication fork may first require ATR–CHK1 signaling, then fork reversal and protection, and finally HR or alternative end joining if the fork collapses. A base lesion can become a replication‐associated DSB when PARP trapping, oxidative stress, or nucleotide imbalance prevents timely repair. This pathway plasticity is therapeutically important because inhibiting one DDR node often changes dependence on another. It is also immunologically important because different repair outcomes produce different substrates for sensing: clean repair is usually silent, whereas mis‐segregated chromosomes, under‐replicated DNA, unresolved R‐loops, and collapsed forks are more likely to produce micronuclei or cytosolic nucleic acids [3, 23, 27, 28, 40, 41].

This integrated view also clarifies why DDR inhibitors should not be grouped only by the lesion they target. PARP inhibitors can function as synthetic‐lethal agents, replication‐fork poisons, PAR metabolism modifiers, and immune modulators. ATR inhibitors can be checkpoint inhibitors, replication‐catastrophe inducers, and radiosensitizers. WEE1 inhibitors are usually described as G2‐M abrogators, but their immunologic effects may depend more on whether they create a synchronized pulse of mitotic failure than on WEE1 inhibition per se. ATM and DNA‐PK inhibitors are both linked to DSB biology, yet ATM perturbation can alter checkpoint signaling and antigen presentation, whereas DNA‐PK perturbation can shape radiation‐induced inflammatory signaling [21, 22, 39, 42, 43, 44, 45].

A broad review of DDR targeting must therefore include both established therapeutic liabilities and less mature immune liabilities. Established examples include PARPi sensitivity in HRD and MSI‐H sensitivity to immune checkpoint blockade. Candidate modifiers include extracellular nucleotide metabolism [37] and replication‐stress/ATR‐linked immune phenotypes [46, 47]. PARPi–ICB studies provide clinical evidence across breast and ovarian cancer settings [48, 49, 50, 51, 52, 53]. Biomarker‐directed ceralasertib studies and preclinical work further illustrate ATRi–ICB development [54, 55, 56, 57]. ARID1A‐linked chromatin and DNA‐repair states provide another immune‐relevant DDR context [58]. Preclinical examples include POLQ inhibition in HR‐deficient tumors, STING agonist rescue of poor endogenous sensing, and epigenetic viral mimicry as a way to create inflammatory vulnerability [59, 60, 61, 62, 63, 64, 65, 66].

DDR pathways are organized around lesion type and cell‐cycle context. HR repairs double‐strand breaks (DSBs) with high fidelity in S and G2 phases and depends on BRCA1, BRCA2, PALB2, RAD51, and Fanconi anemia components. NHEJ and alternative end joining repair DSBs in broader cell‐cycle settings, with DNA‐PK, KU70/80, XRCC4, LIG4, and polymerase theta contributing to pathway choice. MMR corrects replication errors, whereas base and nucleotide excision repair remove small base lesions and bulky adducts. Checkpoint kinases coordinate these repair activities with cell‐cycle progression: ATM senses DSBs, ATR responds to replication protein A‐coated single‐stranded DNA at stalled forks, CHK1 and WEE1 preserve S/G2 checkpoints, and PARP1/2 regulate single‐strand break repair, fork stability and PAR‐dependent stress signaling [1, 2, 3, 4, 5, 6, 7].

Therapeutic DDR targeting exploits either an inherited or acquired repair defect, a lineage‐specific replication‐stress dependency, or a treatment‐induced vulnerability. PARP inhibitors are most effective when HR is impaired, but PARP trapping, fork protection and the immune state also influence response [8, 10, 14, 15, 16, 17, 18, 67]. ATR and WEE1 inhibitors exploit tumors that cannot tolerate S‐phase stress or checkpoint abrogation. WEE1 inhibition can also be used to intensify checkpoint failure or to sequence after PARP inhibition [68]. ATR inhibitors have entered early clinical development [69, 70], while WEE1 inhibitors have additional early clinical and mechanistic support [71, 72, 73]. SETD2 loss, ARID1A deficiency, ATM loss, and SCLC exemplify replication‐stress contexts that can support ATR‐pathway targeting [46, 74, 75, 76, 77]. ATM and DNA‐PK inhibitors may sensitize tumors to radiotherapy and alter antigen presentation or innate inflammatory outputs [21, 22, 39, 42, 43, 44]. These targets therefore differ not only in cytotoxic mechanism, but also in the immune signals they create. Table 1 summarizes the major targetable DDR modules, their immune interfaces, and current clinical maturity.

**TABLE 1:** Major targetable DDR modules and their immune interfaces.

DDR module or state	Main targetable biology	Immune interface	Clinical maturity	Implication for this review
MMR deficiency/MSI‐H [7, 35]	Defective correction of replication errors; frameshift neoantigens; high indel burden.	High neoantigen availability, T‐cell infiltration in many tissues, but variable antigen presentation and immune geography.	Approved/clinically established for ICB in multiple cancers.	Benchmark example showing that DDR genotype can predict immunotherapy, but also that tissue context and immune escape still matter.
Homologous recombination deficiency (BRCA1/2, PALB2, FA pathway) [8, 10, 14, 15, 16, 17, 18]	Impaired high‐fidelity DSB repair, genomic scars, fork‐protection defects; platinum and PARP inhibitor sensitivity.	Micronuclei, cGAS–STING activation, antigen release, PD‐L1 feedback; immune effects depend on STING competence and host‐cell sensing.	Clinically established for PARPi in selected ovarian, breast, prostate and pancreatic cancers; immune‐combination evidence mixed.	Useful anchor for discussing why repair dependency and immune readiness overlap but are not identical.
Replication‐stress dependence (ATR–CHK1–WEE1) [46, 47, 68, 76, 77]	Oncogene‐driven S‐phase stress, stalled forks, checkpoint dependence, and mitotic catastrophe.	Pulsed replication catastrophe can generate micronuclei, IFN signaling and MHC‐I induction; excessive exposure may suppress proliferating lymphocytes.	Early to mid‐stage clinical development; several randomized studies ongoing or reported.	Highlights schedule, dose, and tumor lineage as determinants of immune benefit.
ATM and DNA‐PK controlled DSB signaling [21, 22, 39, 43, 44]	Checkpoint control, NHEJ, radiation response, and DSB persistence.	STING, DNA‐PK–NF‐kappaB, MHC‐I upregulation, macrophage PD‐L1 induction, and radiation‐immune coupling.	Clinical/translational for combinations with radiotherapy or ICB.	Illustrates target‐specific rather than class‐wide DDRi immunology.
PARP/PARG and single‐strand break repair [8, 10, 11, 67]	PAR metabolism, SSB repair, replication fork collapse, and synthetic lethality in HRD.	cGAS–STING activation, immunogenic cell‐death‐like stress, macrophage/Treg remodeling; possible T‐cell fitness effects.	Approved PARPi monotherapy in several indications; combination data heterogeneous.	Provides the richest clinical experience for translating DDR immune priming.
Epigenetically regulated DDR and ERV programs [59, 60, 61, 62]	DNMT, EZH2, PRMT5, and chromatin regulators coordinate repair genes and repeat‐element silencing.	ERV dsRNA activates RIG‐I/MDA5–MAVS viral mimicry; DNA lesions activate cGAS–STING; both converge on IFN programs.	Preclinical/translational, with early combination concepts.	Expands the framework beyond canonical DNA sensors.

*Note*: Clinical maturity labels distinguish established biomarkers from translational or preclinical concepts.

### 2.2 From Genomic Instability to Therapeutic Vulnerability Across Tumor Lineages

Ovarian cancer illustrates the need to separate molecular class, treatment history, and immune state. High‐grade serous ovarian cancer frequently carries HRD and is often initially platinum‐sensitive, but recurrent disease may acquire BRCA reversion mutations, restore RAD51 loading, protect replication forks, or select for immune‐suppressive ascites and macrophage programs. A newly diagnosed HRD tumor may therefore respond differently from a PARPi‐exposed HRD tumor even if both retain a genomic scar. For immune combinations, the relevant question is whether current repair function and current tissue ecology still permit cGAS–STING activation, antigen presentation and effector‐cell access [10, 14, 15, 16, 67, 78, 79, 80].

Breast cancer shows a different pattern. BRCA‐mutant triple‐negative breast cancer can be sensitive to PARPi and may contain baseline immune infiltration, but hormone‐receptor‐positive BRCA‐mutant disease, basal‐like disease, and metaplastic or low‐MHC‐I states differ considerably. This distinction helps explain why early PARPi–ICB activity in selected TNBC cohorts has not automatically translated into broad randomized success. It also supports integrated selection using germline or somatic BRCA status, PD‐L1, TILs, HLA Class I integrity, and dynamic evidence of immune priming rather than relying on one marker [50, 52, 81, 82, 83].

Small‐cell lung cancer and pancreatic cancer define two opposite translational problems. SCLC has abundant replication stress and often high TMB, yet antigen‐presentation repression and rapid treatment resistance can prevent durable immune control. Pancreatic cancer may have actionable HRD in a minority, but dense stroma and myeloid suppression can dominate even when neoantigen load increases. In both settings, the limiting step is not simply whether a DDR vulnerability exists, but whether a therapy can open the immune circuit without destroying the host effector compartment [66, 76, 77, 84, 85, 86, 87, 88, 89].

The immune meaning of DDR defects varies sharply by tumor lineage. MSI‐high colorectal and endometrial cancers often show frameshift neoantigens and clinical sensitivity to PD‐1 blockade, whereas dMMR glioblastoma and heterogeneous MMR loss can behave differently [90, 91, 92, 93, 94]. Recent reviews and disease‐specific studies further show that MSI/MMR context influences immune phenotype and therapeutic response [95, 96, 97, 98, 99, 100, 101]. The foundational clinical studies established sensitivity of MMR‐deficient tumors to PD‐1 blockade [102, 103]. BRCA‐mutant high‐grade serous ovarian cancer may be PARPi sensitive yet immunologically mixed, with macrophages, Tregs, ascites, and acquired HR restoration limiting durable immune control [8, 10, 11, 14, 15, 16, 67]. Triple‐negative breast cancer often contains more pre‐existing immune infiltration than other breast cancer subtypes, but antigen‐presentation defects and variable PD‐L1 expression limit simple biomarker transfer [50, 81, 82].

Pancreatic cancer shows why genomic damage alone is insufficient. HR‐DDR defects may increase TMB and predicted neoantigen load without increasing effector T‐cell density or clonal diversity, consistent with dense stroma and myeloid suppression acting as dominant gates [66, 84, 85, 86, 87, 88]. Small‐cell lung cancer offers a different example: high replication stress and TMB coexist with lineage‐specific MHC‐I repression and rapid immune adaptation, making ATR/WEE1 strategies attractive but schedule‐sensitive [76, 77]. In prostate cancer, BRCA2, ATM, and CDK12 alterations define biologically distinct states rather than a single HRR‐immunogenic category [17, 18]. Table 2 summarizes these lineage‐specific differences.

**TABLE 2:** Lineage‐specific DDR–immune phenotypes and contextual modifiers.

Tumor lineage	Dominant DDR features	Typical immune phenotype	Contextual modifier	Therapeutic implication
High‐grade serous ovarian cancer [14, 15, 16, 67, 78, 79, 80]	Frequent HRD, BRCA1/2 alterations, replication stress, and PARPi sensitivity.	Inflamed and suppressive programs can coexist; macrophages, Tregs, ascites, and acquired HR restoration limit durability.	Platinum sensitivity, HRD assay result, STING competence, fork protection, and prior PARPi exposure.	PARPi remains central, but PARPi–ICB benefit requires biomarker and schedule refinement; WEE1/ATR sequencing is plausible.
Triple‐negative breast cancer [50, 52, 81, 82, 83]	BRCA‐associated HRD in a subset, high replication stress, and genomic instability.	More immune‐infiltrated than hormone‐receptor‐positive disease, but heterogeneous PD‐L1 and antigen‐presentation competence.	Germline BRCA status, PD‐L1, tumor‐infiltrating lymphocytes, basal lineage, and prior chemotherapy.	PARPi or ATR/WEE1 priming may combine with ICB in selected states; negative or mixed Phase III signals argue against unselected use.
Small‐cell lung cancer [76, 77]	Extreme replication stress, TP53/RB1 loss, MYC‐driven S‐phase pressure, and ATR/CHK1 dependence.	Often high TMB but variable antigen presentation and rapid immune adaptation.	MYC status, neuroendocrine lineage, MHC‐I repression, prior platinum/etoposide, and marrow reserve.	ATR/CHK1/WEE1 strategies may be immune‐priming, but host lymphocyte toxicity and timing are central.
Pancreatic ductal adenocarcinoma [66, 84, 85, 86, 87, 88, 89]	HRD in a minority; dense desmoplasia, hypoxia, and stromal control dominate many cases.	TMB/neoantigen increases do not reliably translate into CD8 entry or clonal T‐cell expansion.	Stromal density, myeloid suppression, vascular access, and intact antigen‐presentation machinery.	Repair defects can guide platinum/PARPi; immune conversion likely needs stromal or myeloid cotargeting.
Prostate cancer [17, 18, 42]	HRR alterations in advanced disease; ATM, BRCA2, and CDK12‐associated states differ biologically.	Often immune‐cold; CDK12 and selected HRR states may generate distinctive neoantigen or inflammatory programs.	Lineage plasticity, androgen receptor signaling, bone microenvironment, and prior PARPi/ARSI exposure.	PARPi and radionuclide combinations need biomarkers separating repair dependency from immune accessibility.
Glioma/glioblastoma [21, 35, 91, 92, 93, 94]	MMR deficiency can emerge after alkylators; DNA‐PK and cGAS interactions may drive inflammation.	MMR loss may promote immune suppression rather than a classical MSI‐H inflamed state.	CNS immune ecology, steroid exposure, blood–brain barrier, myeloid dominance, and subclonal MMR loss.	MSI‐H rules from extracranial tumors should not be transferred without tissue‐specific evidence.

*Note*: The same DDR defect can produce different immune states across tumor types.

### 2.3 Contextual Determinants of DDR Immune Output

Another important modifier is lesion topology. A DSB repaired promptly within chromatin differs from a broken chromosome that mis‐segregates into a micronucleus. An R‐loop resolved by helicases and nucleases differs from a persistent transcription–replication conflict that exposes hybrid‐associated nucleic acids. A high TMB tumor with clonal frameshift antigens differs from a tumor with mainly subclonal or poorly presented mutations. These distinctions are often lost when tumors are summarized by broad labels such as DDR‐high, HRD, or genomically unstable. A more useful classification describes the lesion, the processing pathway, the sensing compartment, and the immune barrier that follows.

The same logic applies to dose and treatment field. Local radiotherapy can generate intense damage and antigen release inside one lesion while sparing systemic immune cells; systemic DDR inhibitors may produce lower lesion‐specific damage but broader effects on tumor and host proliferating cells. Metronomic chemotherapy, fractionated radiotherapy, short ATRi pulses, and PARPi run‐in schedules can therefore have different immune consequences even when the same nominal agents are used. This is why scheduling is discussed as a biological variable rather than a logistical detail in Section 4.6.

A central reason DDR‐immunotherapy data are heterogeneous is that immune output depends on modifiers rather than on damage quantity alone. Damage duration is one modifier. A pulsed burst of unrepaired DNA can synchronize antigen release, cGAS–STING activation and chemokine production, whereas chronic low‐level damage may induce IFN tolerance, PD‐L1 feedback, senescence‐associated remodeling, and suppressive myeloid recruitment [10, 19, 32, 34, 104, 105, 106, 107]. Dose intensity is another: excessive DDR inhibition can damage proliferating immune cells or bone marrow before antitumor priming becomes useful [57, 67, 108].

**FIGURE 1:**
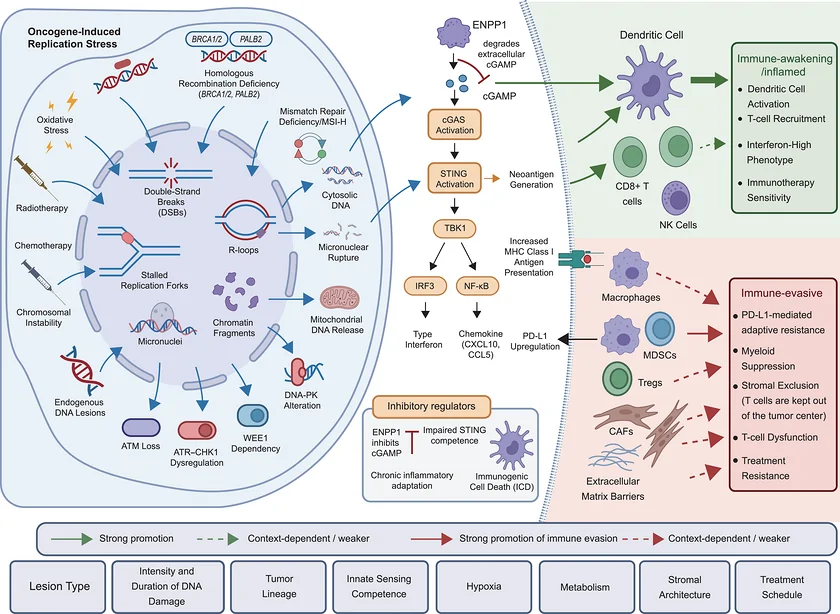
Conceptual framework for targeting the DNA damage response in cancer. The figure maps lesion types and therapeutic stresses to DDR outputs, cytosolic nucleic‐acid sensing, antigen presentation, immune‐cell recruitment, and immune‐evasive remodeling. ENPP1 is positioned in the extracellular compartment to emphasize extracellular cGAMP degradation. Arrow thickness and color intensity indicate strong versus context‐dependent links between DNA‐lesion states and immune‐awakening or immune‐evasive outcomes.

Several contextual variables further modify DDR immune output. Tumor lineage and spatial immune architecture can alter immune accessibility and treatment response [109, 110, 111, 112, 113, 114]. Host context can also contribute, including hypoxia and sex‐associated immune variation [115, 116]. Stromal and matrix remodeling can alter both immune function and DNA‐repair behavior [117, 118, 119]. The composition and location of immune populations, including T cells, B cells, and tertiary lymphoid structures, provide additional context [120, 121, 122, 123, 124, 125, 126]. Hypoxia can further suppress MHC‐I antigen presentation [127]. These variables explain why Section 2.3 is not framed as a simple activating‐versus‐suppressive dichotomy. Instead, it identifies the conditions that shift the balance: acute versus chronic damage, tumor‐intrinsic versus host‐cell sensing, IFN competence versus tolerance, accessible versus excluded immune geography, and preserved versus defective antigen presentation. Figure 1 summarizes this title‐wide framework and positions ENPP1 in the extracellular space, where it degrades cGAMP and contributes to dampening of paracrine STING signaling [33, 37].

## 3 Mechanisms of Damage‐to‐Immunity Conversion

DDR perturbation becomes therapeutically useful only when it is interpreted by tumor and host cells in a way that supports immune recognition. This section follows that conversion process from nucleic‐acid sensing to antigen presentation, checkpoint adaptation, senescence, cell death, myeloid remodeling, and stromal exclusion. Each subsection distinguishes mechanisms that are clinically established from those that remain mainly preclinical or hypothesis‐generating.

### 3.1 Cytosolic Nucleic‐Acid Sensing: Canonical and Parallel Pathways

Beyond canonical cGAS–STING sensing, DNA‐PK and epigenetically derepressed endogenous retroviruses (ERVs) activate parallel cytosolic nucleic‐acid sensing axes—via the DNA‐PK–NF‐κB and RLR–MAVS pathways, respectively—that converge on interferon and inflammatory outputs. Tumor‐intrinsic STING competence is heterogeneous. Some tumors silence STING or downstream IFN genes through methylation, lineage‐specific transcriptional programs, or selection during chronic inflammation. Others retain tumor‐cell STING but fail to recruit dendritic cells or lose antigen‐presentation capacity after the IFN signal. A third group may have weak tumor‐cell sensing but strong host‐cell sensing, in which damaged tumor cells act mainly as a source of DNA, cGAMP, or dying‐cell material. Therapeutic interpretation therefore depends on the sensing compartment: STING agonism may be useful when host‐cell STING remains available, whereas ENPP1 inhibition may be appropriate when extracellular cGAMP transfer is limiting [33, 37, 128, 129, 130, 131]. Checkpoint blockade is most plausible after antigen presentation and T‐cell access are restored.

DNA‐PK and RLR–MAVS pathways provide important escape routes from a cGAS‐only model. DNA‐PK can respond to DNA ends and cooperate with inflammatory transcriptional programs after DSB accumulation, especially in radiation‐rich contexts and glioblastoma models [21]. ERV‐derived dsRNA, in contrast, emerges from chromatin derepression and activates RIG‐I or MDA5 before converging on MAVS, IRF, and NF‐κB outputs [59, 60, 61, 62, 132]. These pathways may compensate when cGAS–STING is weak, but they may also create different cytokine kinetics and toxicity. Therefore, mechanistic studies and trials should specify which nucleic‐acid sensor is engaged rather than reporting only a generic IFN signature.

Paracrine cGAMP biology is especially relevant to solid tumors. cGAMP can pass through gap junctions, extracellular vesicles, or transporter‐dependent routes and can activate STING in dendritic cells or macrophages. ENPP1 limits this process in the extracellular space and can simultaneously generate immunosuppressive nucleotide metabolites. This means that the same irradiated or DDRi‐treated tumor may be immunogenic in an ENPP1‐low setting and muted in an ENPP1‐high setting. It also suggests that biomarkers of extracellular nucleotide metabolism may help explain why radiotherapy or DDRi combinations produce abscopal or systemic immune effects in some models but not others [33, 37, 129].

The chronic STING problem is not merely theoretical. Persistent IFN signaling can select tumor cells with reduced sensor expression, induce negative regulators, reprogram macrophages and T cells, and create a tissue‐protective state that limits acute inflammation. Autophagic STING turnover and endolysosomal sorting are part of normal signal termination, but in tumors they can become mechanisms by which repeated DDRi or radiotherapy pulses lose immune potency. The optimal intervention may therefore be a pulse that reaches the threshold for dendritic‐cell activation but stops before chronic tolerance dominates [133, 134, 135, 136, 137, 138].

The best‐characterized DDR–immune route remains cGAS–STING: cytosolic double‐stranded DNA activates cGAS, cGAS produces 2′‐3′ cGAMP, and STING triggers TBK1–IRF3 and NF‐κB signaling [20, 24, 25, 26]. But DNA‐PK can also cooperate with cGAS or signal through inflammatory routes after DSB accumulation, and ERV‐derived dsRNA can activate RIG‐I and MDA5 through MAVS after epigenetic derepression [21, 59, 60, 61, 62, 132]. These axes are parallel and complementary rather than subordinate branches of one pathway.

A key mechanistic issue is why not all DNA damage activates STING. Recent structural and cell‐biological work shows that cGAS is tightly regulated across nuclear and cytoplasmic compartments. Nuclear cGAS is restrained by nucleosomes, especially interactions with the H2A–H2B acidic patch, and by chromatin‐associated factors such as barrier‐to‐autointegration factor and nuclear lamina‐associated mechanisms [139, 140, 141, 142, 143, 144, 145]. Activation becomes more likely when micronuclei rupture, when nuclear envelopes fail, during mitotic exposure of chromatin, or when DSB processing and chromatin remodeling release cGAS from inhibitory nucleosomal states [23, 27, 28, 139, 140, 141, 142, 143, 144, 145]. Thus, cytosolic DNA abundance is necessary but not sufficient; localization, chromatin binding, cell‐cycle timing, and nuclear‐envelope integrity all gate the signal.

**FIGURE 2:**
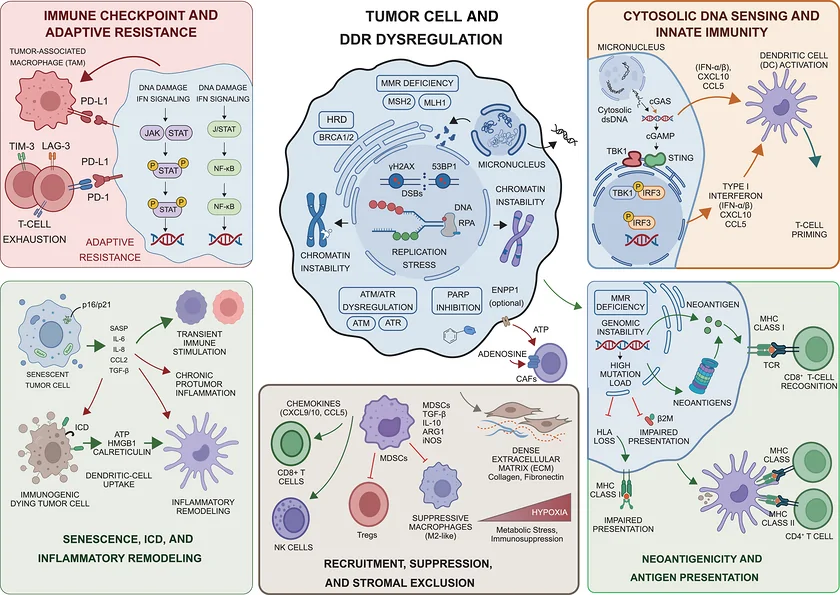
Tumor‐cell and microenvironmental mechanisms linking DDR dysregulation to immunity. The central tumor cell integrates HRD, MMR deficiency, replication stress, micronuclei, chromatin instability, and PARP/ATM/ATR/DNA‐PK‐related signals. Surrounding panels summarize cGAS–STING sensing, neoantigenicity and antigen presentation, immune checkpoint adaptation, senescence and immunogenic cell death, myeloid suppression, stromal exclusion, and hypoxia. Read the four principal quadrants clockwise, starting with innate sensing: (1) innate sensing; (2) antigen presentation; (3) checkpoint adaptation; and (4) senescence/ICD.

The sensing compartment also matters. Tumor cells may lose intrinsic STING signaling yet still release damaged DNA, micronuclei, or cGAMP that host dendritic cells and macrophages can sense. Conversely, tumor cells with intact STING may fail to propagate the signal if extracellular cGAMP is degraded by ENPP1 or if adenosine‐rich metabolism suppresses host immune cells [33, 37, 128, 129]. Chronic activation adds a further limitation. Sustained STING signaling can trigger feedback inhibition at TBK1/IRF3, transcriptional rewiring through STAT1/STAT2, trafficking to endolysosomes, and autophagic degradation involving LC3/GABARAP‐ or p62/SQSTM1‐linked mechanisms [34, 133, 134, 135, 136]. Productive STING signaling is therefore a kinetic and multicellular circuit, not a binary tumor‐cell‐intrinsic marker. Figure 2 provides a mechanism‐level overview of how tumor‐cell DDR dysregulation is interpreted by innate sensing, antigen presentation, checkpoint adaptation, myeloid/stromal compartments, and host immune cells.

### 3.2 Epigenetic–DDR–Immune Circuits and Viral Mimicry

Epigenetic regulators also control antigen presentation and repair gene expression. DNMT inhibition may increase ERV transcripts and IFN‐stimulated genes, but it can also alter HLA, TAP, chemokine, and checkpoint expression. EZH2 inhibition may relieve repression of immune genes, whereas PRMT5 inhibition can impair splicing, DNA repair, and repeat silencing. In a DDR‐targeting framework, these drugs can be viewed as chromatin‐state modifiers that change both the amount of damage and the way that damage is interpreted. The practical biomarker question is whether treatment produces dsRNA/RLR activity, cytosolic DNA/STING activity, MHC‐I induction, or only a nonspecific inflammatory state [59, 60, 61, 62, 132, 146, 147].

Epigenetic regulation links DDR, chromatin state, and innate immunity. DNMT inhibition can derepress endogenous retroviral transcripts and generate dsRNA that activates RIG‐I/MDA5–MAVS signaling. EZH2 inhibition can enhance this viral‐mimicry state by weakening Polycomb‐mediated repression, whereas PRMT5 inhibition can simultaneously impair DNA repair, promote genomic instability, and release ERV‐derived nucleic‐acid signals [59, 60, 61, 62, 132, 148]. This module should be distinguished from cGAS–STING DNA sensing. ERV viral mimicry is primarily an RNA‐sensing program, although it can converge on the same interferon‐stimulated genes and can interact with DNA‐sensing pathways when chromatin damage and repeat derepression coexist.

These mechanisms broaden DDR targeting beyond kinase inhibition. A tumor may be immune cold not because it lacks damage, but because chromatin regulators silence repeat elements, suppress antigen presentation, or maintain repair gene expression. Conversely, epigenetic therapy can create inflammatory signals without a classical HRD or MSI genotype. The translational challenge is to separate useful viral mimicry from chronic nonproductive IFN states and to identify tumors in which epigenetic–DDR therapy creates antigen presentation rather than only cytokine noise.

### 3.3 Neoantigens, Immunopeptidomes, and Antigen‐Presentation Machinery

The immunopeptidome is shaped by both mutational input and processing bias. Peptide display depends on proteasomal processing, peptide transport, ER trimming, HLA loading, and other components of the antigen‐presentation machinery [149, 150, 151, 152, 153]. The immunologic value of candidate neoantigens also depends on mutational burden, clonality, and HLA genotype [154, 155, 156, 157]. HRD‐associated and other mutational signatures influence the variants that are generated [158, 159], while recent work further links MHC‐I‐dependent neoantigen presentation to checkpoint‐blockade response [160]. This distinction is clinically relevant because clonal, high‐quality, properly displayed neoantigens are more likely to support T‐cell recognition than a large number of subclonal or poorly processed candidates.

DDR signaling can also change antigen presentation without changing the mutational repertoire. IFN‐I and IFN‐gamma can induce immunoproteasome subunits, TAP, B2M, and HLA Class I. ATM, ATR, and DNA‐PK pathway perturbation may alter transcription factors, chromatin accessibility, and stress kinases that regulate MHC‐I. Conversely, chronic IFN pressure can select for JAK1/2 loss, B2M loss, or HLA‐I LOH. A tumor can therefore move from antigen‐rich to antigen‐invisible during treatment. This is a strong argument for paired baseline and on‐treatment antigen‐presentation assessment in DDRi–ICB trials [43, 65, 112, 149, 150, 151, 152, 153, 161, 162].

Spatial antigen presentation is equally important. HLA Class I expression on tumor cells near invasive margins may not predict killing if the tumor core is HLA‐low or separated by macrophage‐rich stroma. Conversely, a low bulk HLA signal can hide small immune‐reactive niches. Spatial transcriptomics and multiplex pathology can identify whether CD8+ cells are in contact with HLA‐positive malignant cells or are trapped in fibroblast and myeloid compartments. For DDR‐targeted immunotherapy, this distinction may be more predictive than global TMB or a bulk IFN score [90, 112, 113, 114, 163, 164].

DDR defects can alter tumor antigenicity quantitatively and qualitatively. MMR deficiency produces insertion–deletion mutations and frameshift peptides [35, 95, 102, 103]. HRD and APOBEC activity create distinctive mutational signatures that can contribute to heterogeneous neoantigen repertoires [158, 159, 165, 166]. However, neoantigen quantity does not guarantee presentation. The antigen‐processing machinery must generate peptides in the proteasome or immunoproteasome, transport them into the endoplasmic reticulum through TAP1/2, trim them through ERAP1/2, load them onto HLA Class I with beta‐2 microglobulin and peptide‐loading components, and maintain transcriptional regulation through NLRC5 and IFN–JAK signaling [149, 150, 151, 152, 153].

**FIGURE 3:**
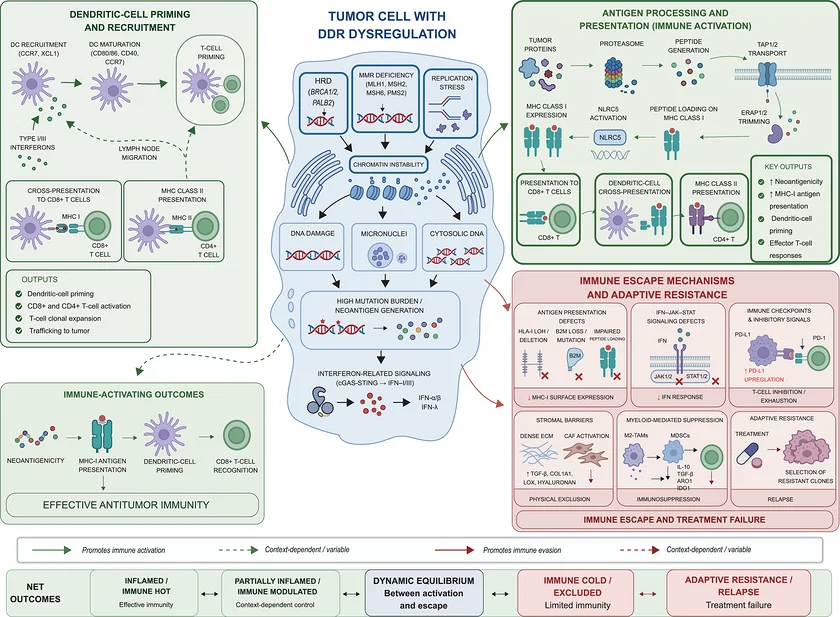
DDR dysregulation, antigen presentation, and immune escape. DDR defects can increase mutational burden, interferon signaling, dendritic‐cell priming, cross‐presentation, and MHC‐I‐dependent T‐cell recognition. Productive immunity can be interrupted by HLA‐I loss of heterozygosity, B2M or TAP defects, impaired IFN–JAK–STAT signaling, checkpoint induction, myeloid suppression, stromal barriers, and selection of resistant clones.

DDR perturbation can influence this machinery directly. ATM inhibition has been reported to increase MHC‐I expression in model‐dependent and dose/schedule‐dependent settings [22, 43]. IFN‐I signaling after cytosolic DNA sensing can induce HLA Class I, B2M, TAP, and immunoproteasome genes, while epigenetic therapies can restore or repress antigen‐presentation programs depending on chromatin context [43, 59, 60, 61, 62, 165]. Immune escape can interrupt this link through HLA‐I loss of heterozygosity, B2M inactivation, TAP/ERAP defects, or other antigen‐presentation failures [149, 150, 151, 152, 153, 167]. Spatial exclusion of CD8+ T cells provides a distinct barrier even when antigen‐presentation machinery is retained [112, 113, 114]. For biomarker selection, therefore, TMB and HRD scars should be paired with evidence that antigens can actually be displayed and encountered. Figure 3 expands this antigen‐presentation and immune‐escape layer, highlighting how neoantigen generation can be uncoupled from effective T‐cell recognition.

### 3.4 Checkpoint Adaptation, Senescence, and Immunogenic Cell Death

Checkpoint adaptation should also be interpreted beyond PD‐L1. TIM‐3, LAG‐3, TIGIT, VISTA, adenosine receptors, macrophage checkpoints, and suppressive cytokines can all rise after damage‐induced inflammation. The right partner for a DDR inhibitor may therefore differ by tissue state: PD‐1/PD‐L1 blockade may suit a T‐cell‐inflamed and PD‐L1‐adapted lesion, whereas myeloid or adenosine targeting may be more rational when DDR therapy mainly recruits macrophages or amplifies extracellular nucleotide metabolism. This is one reason that broad DDRi plus PD‐1/PD‐L1 trials can miss biology that is visible in smaller mechanistically enriched cohorts [10, 32, 33, 38, 39, 168].

Inflammatory DDR signaling frequently induces negative feedback. DNA damage can upregulate PD‐L1 through ATM/ATR/CHK1‐associated stress, IFN‐I transcriptional programs, NF‐κB signaling, and posttranscriptional mechanisms [32, 34, 38, 82, 169]. In this setting, PD‐L1 is not simply a resistance marker; it may be a pharmacodynamic sign that the tumor has become more visible and is activating adaptive suppression. This logic supports DDRi–ICB combinations but also explains why they fail when antigen presentation, T‐cell access, or host‐cell fitness is not preserved.

Persistent damage also induces senescence and immunogenic cell‐death‐like states. Senescent cells can release cytokines, chemokines, proteases, and matrix‐remodeling factors that recruit immune cells, but prolonged senescence‐associated secretory programs can support fibrosis, stemness, and macrophage skewing [104, 105, 106, 170]. Immunogenic cell death after DNA‐damaging agents, prodrugs, ferroptosis‐associated ER stress, or PARP1‐targeted photoimmunotherapy can release danger signals and antigens, but only when the form of death remains interpretable by host immunity [171, 172, 173, 174, 175]. Timing is again central: transient damage may create immune surveillance, whereas unresolved stress can build chronic inflammatory tolerance.

### 3.5 Myeloid, Stromal, and Metabolic Interpretation of DDR Signals

Basic myeloid biology also influences pharmacodynamic interpretation. A rise in interferon‐stimulated genes could reflect dendritic‐cell activation and cross‐priming, but it could also reflect macrophage‐dominant inflammation that limits T‐cell function. Increased PD‐L1 may occur in malignant cells, macrophages, or both. Increased chemokines can recruit effector cells or suppressive myeloid cells depending on the chemokine mix and vascular context. For this reason, future studies should report the cellular source and spatial distribution of DDR‐induced immune signals whenever possible rather than treating bulk RNA expression as sufficient [39, 90, 114, 128].

The tumor immune microenvironment (TIME) determines whether DDR‐derived inflammatory cues become productive. Dendritic cells can convert tumor‐derived DNA or cGAMP into cross‐priming, whereas macrophages can convert the same cues into PD‐L1 expression, adenosine production, angiogenic remodeling, and T‐cell suppression [39, 90, 128, 129, 176, 177]. ENPP1 is particularly important because it hydrolyzes extracellular cGAMP and can promote an immunosuppressive nucleotide‐metabolism state [33, 37].

Stromal and metabolic features further filter DDR output. Hypoxia alters repair‐pathway choice, increases plasticity, and can suppress immune effector function [115, 118, 178]. Cancer‐associated fibroblasts can enhance repair and treatment resistance, as illustrated by CXCL14‐driven nucleotide excision repair in bladder cancer [117]. Dense extracellular matrix, poor vascular access, and macrophage‐rich corridors can trap CD8+ T cells away from malignant cells even when bulk IFN or MHC‐I signals are present [109, 112, 114, 120, 121]. This subsection deliberately focuses on basic mechanisms; the clinically oriented resistance implications are revisited in Section 4.8.

## 4 Therapeutic Targeting of DDR–Immune Biology

Therapeutic DDR targeting must now be interpreted across three axes: tumor‐cell lethality, immune priming, and host immune‐cell tolerance. This section reviews DDR inhibitor classes, immune combinations, STING agonists, cellular immunotherapy, pharmacologic scheduling, and clinical evidence. It emphasizes that PARP, ATR, WEE1, ATM, and DNA‐PK inhibitors are not interchangeable immunologic agents.

### 4.1 Target‐Specific Immune Profiles of DDR Inhibitors

An additional distinction is target engagement versus immune engagement. A DDR inhibitor may clearly increase gamma‐H2AX, phospho‐CHK1 suppression, or replication stress while failing to increase antigen presentation or T‐cell infiltration. Conversely, a modest level of DNA damage may be immunologically effective if it occurs in a STING‐competent, dendritic‐cell‐rich, and spatially accessible tumor. Pharmacodynamic endpoints should therefore be layered: first confirm target inhibition, then confirm damage processing, then confirm innate sensing, antigen presentation, and immune‐cell movement. Without this hierarchy, a negative clinical trial cannot distinguish target failure from immune‐conversion failure.

PARP inhibitors, ATR inhibitors, WEE1 inhibitors, ATM inhibitors, and DNA‐PK inhibitors create different damage states. PARPi trap PARP on DNA, collapse forks and are most effective in HRD, but they may also activate STING and induce adaptive immunity [8, 10, 11, 67, 81, 179, 180, 181]. ATRi collapse replication‐stress tolerance and can create micronuclei and IFN‐I signaling, but they also act on proliferating immune cells because lymphocytes require intact replication checkpoints during clonal expansion [46, 47, 57, 76, 77]. WEE1 inhibitors force damaged cells through mitosis, ATM inhibitors can enhance MHC‐I and STING signaling in model‐dependent and scheduling‐dependent contexts, and DNA‐PK inhibitors may be especially relevant in radiation‐immune coupling [21, 22, 39, 42, 43, 44, 68]. Table 3 compares the immune‐modulatory profiles, liabilities, companion biomarkers, and representative combination concepts of major DDR inhibitor classes.

**TABLE 3:** Comparative immune‐modulatory profiles of representative DDR inhibitor classes.

DDRi class	Immune‐activating pathway	Immune‐suppressive or host‐cell liability	Companion biomarker	Representative combination concept
PARP inhibitors [8, 10, 67, 81, 82, 179]	Cytosolic DNA, micronuclei, cGAS–STING activation, antigen release, and ICD‐like stress in HRD or PARP‐trapping‐sensitive tumors.	HR restoration, fork protection, PD‐L1 feedback, altered macrophage/Treg balance, and possible T‐cell metabolic/DNA‐damage stress.	BRCA1/2 or HRD, RAD51 foci, PARP trapping sensitivity, STING competence, baseline CD8/TAM balance.	PARPi + PD‐1/PD‐L1; PARPi followed by WEE1i/ATRi; PARPi + STING agonist in resistant HRD models.
ATR inhibitors [46, 47, 57, 76, 77]	Collapse of replication‐stress tolerance, micronuclei formation, IFN‐I signaling, and enhanced radiosensitization.	Direct effects on proliferating CD8+ T cells and hematopoiesis; schedule‐dependent immunosuppression if exposure overlaps T‐cell expansion.	Replication‐stress score, ATM/SETD2 loss, phospho‐CHK1, micronuclei, early IFN response.	ATRi + durvalumab; RT → ATRi → ICB; ATRi with cellular therapy after tumor priming but before T‐cell expansion.
WEE1 inhibitors [68, 71, 72, 73]	G2‐M checkpoint abrogation, mitotic catastrophe, inflammatory damage pulses, and possible MHC‐I/chemokine induction.	Dose‐limiting marrow/GI toxicity, broad cell‐cycle stress and rapid adaptive checkpoint signaling.	CCNE1 amplification, TP53 deficiency, replication stress, on‐treatment gamma‐H2AX, and cytokine kinetics.	PARPi → WEE1i sequencing; WEE1i + PD‐1 blockade in replication‐stress‐high tumors.
ATM inhibitors [22, 39, 43]	Model‐dependent STING activation, MHC‐I upregulation and radiosensitization.	Macrophage PD‐L1 induction under irradiation, persistent cold phenotypes in some ATM‐deficient cancers and normal‐tissue sensitivity.	ATM pathway loss/activity, MHC‐I inducibility, myeloid PD‐L1, spatial CD8 access.	ATMi + RT + PD‐L1 blockade; ATMi with myeloid/checkpoint cotargeting.
DNA‐PK inhibitors [21, 42, 44]	DSB persistence, DNA‐PK–NF‐kappaB inflammatory signaling and stronger radiation‐immune coupling.	Normal‐tissue radiosensitization, compensatory innate suppression, and fractionation‐dependent toxicity.	DNA‐PK activity, radiation‐induced IFN, cGAMP turnover, spatial immune redistribution.	DNA‐PKi + focal RT + ICB or radionuclide‐based immune priming.

### 4.2 PARP Inhibitors, Immune Checkpoint Blockade, and Mixed Late‐Stage Signals

The clinical development of PARPi–ICB combinations has also revealed the importance of comparator choice. A single‐arm signal in platinum‐resistant ovarian cancer or TNBC can be encouraging, but randomized maintenance settings test whether adding PARPi to an already active chemo‐immunotherapy platform improves durable outcomes beyond continued chemotherapy or standard maintenance. KEYLYNK‐009 and FIRST illustrate that biologic rationale does not guarantee late‐stage superiority. These studies should not be read as disproving DDR immune modulation; rather, they show that the incremental value of PARPi–ICB depends on tumor state, previous treatment, maintenance comparator, and whether the combination creates new immune biology rather than overlapping toxicity [48, 49, 50, 51, 52, 53].

Resistance to PARPi can be immune‐relevant as well as repair‐relevant. BRCA reversion mutations restore HR and reduce cytosolic DNA generation. Fork‐protection restoration may reduce replication‐associated micronuclei. Altered PAR metabolism can change inflammatory signaling. Inflammatory pressure can select for antigen‐presentation defects or myeloid exclusion. These mechanisms imply that post‐PARPi tumors should not simply be labeled PARPi‐resistant; they should be re‐profiled for current HR function, current sensing competence and current TIME composition [10, 67, 78].

PARPi remain the best clinically developed DDR inhibitors, but their role as immune‐combination partners should be stated cautiously. Preclinical studies show that PARPi can increase cytosolic DNA, cGAS–STING signaling, CD8+ T‐cell recruitment, PD‐L1 expression, and immunogenic stress, particularly in HRD models [8, 10, 11, 81, 82, 169, 179]. Clinically, PARPi monotherapy has transformed care for selected ovarian, breast, prostate, and pancreatic cancers [12, 13, 14, 15, 16, 17, 18]. PARPi–ICB combinations, however, have produced heterogeneous results across Phase I/II and randomized studies [48, 49, 50, 51, 52, 53]. It is therefore more accurate to say that PARPi provide the most mature clinical platform for exploring DDR immune modulation, not the clearest proof that DDRi–ICB combinations are broadly effective.

Several lessons emerge. First, HRD or BRCA mutation enriches for repair dependency but does not guarantee immune accessibility. Second, PD‐L1 upregulation after PARPi may justify checkpoint blockade but can also reflect adaptive resistance. Third, PARPi may affect host T cells directly, including DNA damage and metabolic fitness in settings where T cells are actively proliferating [108]. Fourth, prior platinum or PARPi exposure can restore HR, alter fork protection, and change immune composition. Future PARPi combinations should therefore integrate HRD, RAD51 function, STING competence, antigen presentation, CD8 geography, and dynamic toxicity monitoring rather than relying on BRCA status alone.

### 4.3 ATR, WEE1, ATM, and DNA‐PK Strategies

For ATR inhibitors, the most important design variable may be intermittent exposure. Tumor cells with high replication stress can accumulate lethal or immunogenic damage after a short ATRi pulse, but proliferating T cells also use ATR to maintain genome integrity during expansion. Preclinical evidence that CD8+ T‐cell suppression can reverse after drug withdrawal supports schedules that separate tumor priming from immune expansion. Clinically, this could mean short ATRi windows after radiotherapy, before ICB, or between cellular therapy expansion phases, with blood lymphocyte proliferation and T‐cell DNA damage used as safety‐pharmacodynamic readouts [47, 54, 55, 56, 57, 108, 182].

ATM and DNA‐PK inhibition also require context‐specific schedules. In radiotherapy combinations, fraction size and total dose determine whether cytosolic DNA accumulates or is degraded by nucleases such as TREX1. ATM inhibition may enhance MHC‐I and STING signals in tumor cells but may also increase PD‐L1 in macrophages under irradiated conditions. DNA‐PK inhibition can increase DSB persistence and inflammatory signaling, but radiosensitization of normal tissue can limit dose. These examples support small, biomarker‐rich trials that vary radiation sequence and fractionation rather than one‐size‐fits‐all combinations [21, 22, 29, 30, 31, 39, 43, 44].

ATR inhibition is attractive in replication‐stress‐high cancers such as small‐cell lung cancer, SETD2‐deficient renal cancer, ARID1A‐deficient tumors, and tumors with ATM loss [46, 47, 69, 74, 76, 77, 179, 183]. ATRi can increase micronuclei and IFN signaling and may sensitize tumors to radiotherapy or PD‐L1 blockade. Yet ATRi can also suppress proliferating CD8+ T cells during exposure; preclinical data suggest this effect can be reversed when dosing is scheduled to spare the T‐cell expansion phase [57]. Thus, ATRi may be best used as short tumor‐priming pulses before or between immune‐effector phases rather than as continuous immune pressure.

WEE1 inhibitors, ATM inhibitors and DNA‐PK inhibitors provide complementary strategies. WEE1 inhibition may be useful after PARPi priming or in CCNE1‐high and TP53‐deficient states, but marrow and gastrointestinal toxicity can narrow the immune‐priming window [68, 71, 72, 73, 184]. ATM inhibition can increase MHC‐I and activate STING in some models, but this effect is model‐dependent and dose/schedule‐dependent, and macrophage PD‐L1 induction during irradiation may blunt benefit [22, 39, 43]. DNA‐PK inhibitors may enhance DSB persistence and inflammatory radiation responses, but normal tissue radiosensitization and innate feedback require careful fractionation and field design [21, 42, 44].

### 4.4 STING Agonists and Epigenetic–DDR Combinations

The STING agonist field also teaches a delivery lesson. Systemic STING activation can be limited by toxicity, whereas intratumoral delivery may generate strong local inflammation but is feasible only for accessible lesions and may not affect occult metastases. DDR inhibitors and radiotherapy may provide tumor‐localized endogenous DNA damage, while STING agonists amplify sensing in host cells. The ideal setting may therefore be a tumor with poor endogenous STING activation despite adequate damage, preserved host APC STING, and a delivery strategy that avoids systemic cytokine toxicity [63, 64, 65, 185].

STING agonists provide a logical partner for DDR inhibitors when damage is generated but innate sensing is insufficient. Agents such as MIW815/ADU‐S100 and SB11285 were developed to activate STING within the tumor microenvironment, often through intratumoral administration, and early clinical studies have shown feasibility but modest single‐agent or dual‐checkpoint activity [63]. Preclinical studies combining STING agonism with PARP inhibition suggest that exogenous STING activation can overcome weak endogenous cGAMP signaling, increase dendritic‐cell activation, and recruit cytotoxic lymphocytes, including in models of PARPi resistance [64, 65].

Epigenetic–DDR combinations follow a related but distinct principle. DNMT, EZH2, or PRMT5 inhibition can derepress ERVs, activate RIG‐I/MDA5–MAVS viral mimicry, alter DDR gene expression and potentially prime ICB [59, 60, 61, 62, 132, 186]. These combinations should not be described as simply boosting cGAS–STING. Their immune effect may be RNA‐sensing dominant, DNA‐sensing dominant or mixed depending on chromatin context and damage burden. Biomarkers should therefore separate ERV‐dsRNA signatures from cytosolic DNA/cGAMP signatures.

### 4.5 DDR Inhibitors and Cellular Immunotherapy

Cellular therapy combinations also raise questions that are specific to antigen type. TCR‐T and TIL approaches require HLA‐restricted peptide presentation, so DDR‐induced MHC‐I and immunopeptidome remodeling may be directly useful. CAR‐T cells recognize surface antigens independently of HLA, but DDRi or radiotherapy may still help by increasing chemokines, vascular access, and inflammatory adhesion signals. At the same time, antigen heterogeneity and stromal exclusion remain important barriers. Trials should therefore define whether DDRi is intended to improve antigen display, trafficking, persistence, or local inflammatory support, because each goal requires a different timing and biomarker plan.

DDRi combinations with cellular immunotherapy are an emerging frontier. The rationale is that DDR inhibition can increase MHC‐I expression, antigen density, chemokines such as CXCL10 and CCL5, and local inflammation that may improve trafficking and function of CAR‐T, TCR‐T, or TIL products. ATRi, PARPi, WEE1i, or ATMi could be used to prime tumor cells before adoptive transfer, while radiotherapy can create focal antigen release and vascular remodeling that supports cell entry.

The risk is that DDRi also damage the therapeutic immune cells. CAR‐T, TCR‐T, and TIL products undergo rapid proliferation, DNA replication, and metabolic remodeling; continuous ATRi or PARPi exposure during this phase could reduce expansion, persistence, or killing capacity [57, 108]. A rational design would separate tumor priming from immune‐cell expansion: short DDRi exposure before cell infusion, washout or intermittent dosing during expansion, and checkpoint or cytokine support after antigen presentation has increased. Early clinical translation should include cell‐product fitness, persistence and exhaustion markers as mandatory pharmacodynamic endpoints.

### 4.6 Pharmacologic Scheduling: Why Sequence Matters

The most informative schedules will probably be adaptive rather than fixed. For example, a trial could administer a short DDRi or radiotherapy priming phase, measure cytosolic DNA, IFN signaling, MHC‐I, and PD‐L1 after 24–72 h, and add ICB only if immune conversion occurs. A second biopsy or blood panel could assess CD8 expansion and myeloid compensation after one or two cycles, guiding whether to continue, add a myeloid/stromal partner, or de‐escalate DDRi exposure to protect lymphocytes. This approach treats schedule as a testable biological hypothesis rather than as a standard dosing convention.

Scheduling matters because repair inhibition, innate sensing, antigen presentation, checkpoint induction, and T‐cell expansion peak at different times. DNA repair pathway engagement occurs within minutes to hours after damage. Micronuclei and cytosolic DNA often emerge after cell‐cycle progression or mitotic failure, creating a 6‐ to 24‐h window for cGAS–STING activation. IFN‐stimulated genes, chemokines, and PD‐L1 may peak over the next 24–72 h. Dendritic‐cell cross‐priming and T‐cell infiltration often require several days. If a DDR inhibitor is given continuously through the T‐cell expansion phase, it may suppress the same immune cells it is intended to recruit [57, 67, 108].

This temporal logic supports several strategies. Radiotherapy followed by ATR inhibition can exploit radiation‐induced replication and checkpoint stress while allowing innate sensing to develop. PARPi followed by WEE1 inhibition may create a second damage pulse after fork collapse, but dose intensity must avoid marrow suppression [68]. DDRi followed by ICB may be preferable when PD‐L1 induction lags behind DNA damage, whereas concurrent DDRi–ICB may be appropriate when an inflamed but checkpoint‐adapted state already exists. Dynamic sampling is therefore not optional; it is the only way to determine whether a schedule has produced immune priming or adaptive tolerance.

### 4.7 Clinical Evidence for DDRi and ICB Combinations

Evidence levels should remain explicit when interpreting these trials. MEDIOLA and TOPACIO provide early evidence that PARPi–ICB combinations are feasible and can produce responses in selected populations, but they do not identify a universal biomarker [48, 49, 50, 51]. KEYLYNK‐009 and FIRST provide stronger randomized evidence that broad PARPi–ICB strategies may have modest or no incremental benefit in some late‐stage settings [52, 53]. HUDSON and ceralasertib studies support ATRi–ICB as a biomarker‐directed concept in selected immune‐resistant settings [54, 55]. These combination strategies remain exploratory compared with established MSI‐H/dMMR checkpoint blockade [102, 103] and approved PARPi indications [14, 15, 16, 17, 18].

Clinical evidence now spans early single‐arm studies and randomized trials. MEDIOLA evaluated olaparib plus durvalumab in germline BRCA‐mutated metastatic breast cancer and in ovarian cancer cohorts [48, 51]. TOPACIO/KEYNOTE‐162 evaluated niraparib plus pembrolizumab in recurrent platinum‐resistant ovarian cancer and metastatic triple‐negative breast cancer [49, 50]. KEYLYNK‐009 tested maintenance pembrolizumab plus olaparib after induction therapy in advanced triple‐negative breast cancer, while FIRST/ENGOT‐OV44 tested dostarlimab with chemotherapy followed by dostarlimab plus niraparib in primary advanced ovarian cancer [52, 53]. ATR inhibition has entered immune combinations through ceralasertib plus durvalumab, including the HUDSON biomarker‐directed NSCLC platform and anti‐PD‐1‐refractory melanoma [54, 55]. Table 4 summarizes representative studies and the level of evidence.

**TABLE 4:** Representative clinical studies of DDR‐directed therapy combined with immune checkpoint blockade.

Trial/regimen	Tumor type and biomarker	Treatment schedule	Activity signal	Safety and evidence level
MEDIOLA breast: olaparib + durvalumab [48]	HER2‐negative metastatic breast cancer with germline BRCA mutation.	Olaparib 300 mg twice daily run‐in for 4 weeks, then olaparib plus durvalumab 1.5 g every 4 weeks.	12‐week disease control was high in the Phase I/II cohort; responses supported feasibility of PARPi–ICB in gBRCA disease.	Grade ≥3 adverse events included anemia, neutropenia, and pancreatitis; early clinical evidence.
MEDIOLA ovarian: olaparib + durvalumab ± bevacizumab [51]	Platinum‐sensitive relapsed ovarian cancer, including BRCA‐mutated and broader cohorts.	Olaparib and durvalumab, with an anti‐VEGF‐containing arm in expansion cohorts.	Clinical activity was observed, with stronger rationale where HRD and immune/vascular modulation overlapped.	Grade ≥3 adverse events were common but generally manageable; Phase II translational evidence.
TOPACIO/KEYNOTE‐162 ovarian: niraparib + pembrolizumab [49]	Recurrent platinum‐resistant ovarian carcinoma, not restricted to BRCA mutation.	Niraparib daily plus pembrolizumab every 3 weeks.	Objective responses occurred in a minority but disease control was seen beyond BRCA‐mutated tumors.	Hematologic toxicity, especially anemia and thrombocytopenia, was frequent; Phase I/II evidence.
TOPACIO/KEYNOTE‐162 TNBC: niraparib + pembrolizumab [50]	Advanced/metastatic triple‐negative breast cancer; exploratory enrichment by tumor BRCA mutation and PD‐L1.	Niraparib daily plus pembrolizumab every 3 weeks.	Responses were higher in tumor BRCA‐mutated disease than in wild‐type disease; overall activity remained modest.	No unexpected safety signal, but hematologic and immune‐related events require monitoring; Phase I/II evidence.
KEYLYNK‐009: pembrolizumab + olaparib maintenance [52]	Locally recurrent inoperable or metastatic TNBC after induction chemo‐immunotherapy.	Maintenance pembrolizumab plus olaparib compared with pembrolizumab plus chemotherapy after response/stable disease.	Primary endpoint was not met; benefit was similar to the control maintenance approach.	Late‐stage randomized evidence showing that PARPi–ICB is not universally superior.
FIRST/ENGOT‐OV44: dostarlimab + chemotherapy → dostarlimab + niraparib [53]	Newly diagnosed Stage III–IV epithelial ovarian cancer.	Carboplatin–paclitaxel with or without dostarlimab followed by niraparib maintenance with or without dostarlimab.	PFS benefit was statistically significant but clinically modest, with no OS improvement at reporting.	Toxicity consistent with known chemotherapy, ICB, and PARPi profiles; randomized Phase III evidence.
HUDSON module: ceralasertib + durvalumab [54]	Immunotherapy‐pretreated advanced NSCLC; exploratory ATM‐altered subgroup.	Durvalumab with intermittent oral ceralasertib.	Higher response and longer PFS/OS than other biomarker‐matched modules; ATM‐altered tumors appeared enriched.	Grade ≥3 treatment‐related adverse events were acceptable; biomarker‐directed Phase II evidence.
Ceralasertib + durvalumab in anti‐PD‐1‐refractory melanoma [55]	Advanced melanoma after anti‐PD‐1 failure.	Intermittent ceralasertib plus durvalumab.	Clinically meaningful responses were reported in a single‐arm Phase II study.	Hematologic adverse events were common but manageable; early clinical evidence.

*Note*: Outcome terms are summarized to emphasize translational interpretation rather than replace the original trial reports.

### 4.8 Resistance and Translational Challenges

A practical resistance framework should ask four questions at progression. First, is the target still relevant, or has repair dependency been restored? Second, does treatment still generate damage substrates such as micronuclei or cytosolic DNA? Third, are sensing, IFN signaling, and antigen presentation intact? Fourth, has the tissue become physically or metabolically inaccessible to immune cells? Answering these questions requires tissue whenever possible, but ctDNA, blood immune profiling, and imaging of lesion‐specific responses can provide complementary information when repeat biopsy is not feasible.

Clinical resistance can arise at every step of the DDR–immune chain. The intended DDR target may be bypassed by HR restoration, reversion mutations, fork protection, alternative end joining, drug efflux, or checkpoint rewiring [67, 78, 187, 188, 189]. Damage may fail to become inflammation because tumor cells lose STING competence, cGAMP is degraded by ENPP1, cGAS remains nucleosome‐restrained, or chronic IFN signaling becomes tolerized [33, 37, 134, 135, 136, 139, 140, 141, 142, 143]. Inflammation may fail to become cytotoxic immunity because antigen presentation is lost through HLA‐I, B2M, TAP, or related defects [149, 150, 151, 152, 153, 167]. CD8+ cells can also be excluded by macrophage, fibroblast, hypoxic, or matrix barriers [112, 114, 115, 117, 127].

**FIGURE 4:**
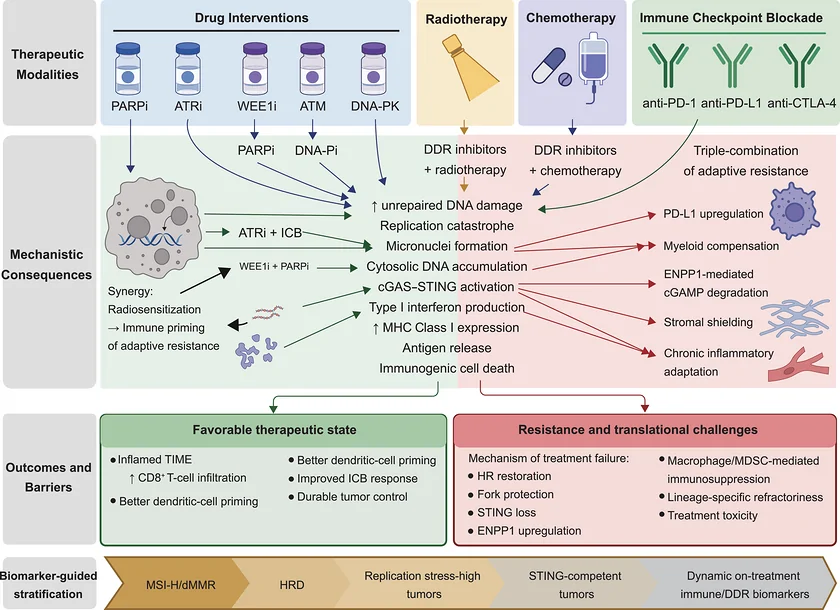
Therapeutic modalities and translational consequences of DDR targeting. PARP, ATR, WEE1, ATM, and DNA‐PK pathway inhibitors can be combined with radiotherapy, chemotherapy, or immune checkpoint blockade to increase unrepaired DNA damage, micronuclei, cytosolic DNA, cGAS–STING activation, Type I interferon, antigen release, and immunogenic cell death. The same interventions can promote PD‐L1 upregulation, myeloid compensation, ENPP1‐mediated cGAMP degradation, stromal shielding, chronic inflammatory adaptation, and treatment failure.

Toxicity is another resistance‐like barrier because it prevents delivery of the biologically optimal schedule. Myelosuppression, gastrointestinal toxicity, and host lymphocyte DDR dependence can narrow the therapeutic index for ATRi, WEE1i, and PARPi combinations [57, 67, 108]. Negative trials should therefore be interpreted carefully: they may reflect inadequate patient selection, wrong schedule, insufficient immune conversion, excessive host‐cell suppression, or a tumor lineage in which the downstream sensing circuit is absent. Figure 4 summarizes the therapeutic design logic across DDR‐directed drug interventions, radiotherapy, chemotherapy, and immune checkpoint blockade.

## 5 Biomarkers for Patient Stratification

Because the preceding sections show that the same DDR lesion can lead to different immune consequences depending on damage processing, sensing competence, and tissue context, the transition from mechanism to therapy cannot rely on genotype alone. Section 5 therefore focuses on biomarkers for patient stratification: they are needed to identify patients in whom a targetable DDR dependency is both present and capable of being converted into productive antitumor immunity. In this framework, a useful biomarker does not simply state whether a DDR lesion exists; it estimates whether that lesion can be exploited as a drug vulnerability and whether the downstream immune‐conversion machinery is intact. This section separates evidence levels and proposes a practical modular framework.

### 5.1 Distinguishing Levels of Evidence

A simple four‐level evidence language can improve translational clarity. Level 1 includes approved or practice‐changing markers such as MSI‐H/dMMR for PD‐1 blockade and HRD/BRCA for PARPi in specific indications. Level 2 includes randomized or prospective clinical evidence that is not yet broadly practice‐changing, such as selected DDRi–ICB combinations. Level 3 includes clinical‐translational or pharmacodynamic markers such as RAD51 foci, ATM alteration in ATRi combinations, or spatial immune markers. Level 4 includes preclinical or hypothesis‐generating concepts such as STING agonist rescue, ENPP1 selection, or cellular therapy priming. Tables and trial designs should state which level is being used.

Evidence should be interpreted hierarchically. MSI‐H/dMMR as a predictor of response to PD‐1 blockade is clinically established across several tumor types, although important exceptions remain [95, 96, 97, 98, 99, 102, 103, 190]. BRCA/HRD biomarkers are clinically established for PARPi in selected cancers but are less reliable as stand‐alone immunotherapy predictors [8, 10, 11, 12, 13, 14, 15, 16, 17, 18]. ATRi, WEE1i, ATMi, DNA‐PKi, and STING agonist combinations are supported by preclinical, translational, and early clinical evidence, but most are not yet practice‐changing [42, 43, 46, 54, 63, 68, 69]. Evidence labels should therefore accompany biomarker tables and trial designs.

### 5.2 Genomic Biomarkers

Genomic scars need special caution. HRD scores and mutational signatures record historical repair deficiency but may not reflect current HR function after clonal selection or reversion. TMB records mutational burden but not clonality, HLA binding, antigen processing, or immune access. MSI‐H often predicts ICB sensitivity, but heterogeneous MMR loss, lineage‐specific immune ecology, and CNS context can change the outcome. Therefore, genomic biomarkers should be combined with functional assays whenever the therapeutic decision depends on current repair state or immune conversion [88, 94, 100, 101, 158, 159, 187, 188, 189, 191].

Genomic biomarkers remain the entry point for many trials. MSI‐H/dMMR provides a clinically established immunotherapy biomarker [95, 102, 103]. BRCA1/2, PALB2, Fanconi anemia genes, ATM, CDK12, and SETD2 can define repair dependencies or replication‐stress vulnerabilities in specific settings [17, 18, 46, 187, 188, 189]. HRD scars, APOBEC signatures, and TMB capture broader genomic states but do not directly measure current immune readiness [154, 158, 159, 165, 166]. Other emerging genomic correlates, including CHEK2 deficiency, TTN mutation, and germline DNA‐repair/immune‐metabolic patterns, remain exploratory and context dependent [192, 193, 194]. But genotype alone cannot establish immune readiness. Pancreatic HR‐DDR defects can raise TMB and neoantigen load without increasing CD8 density, and dMMR glioblastoma may shift toward immune suppression rather than canonical MSI‐H sensitivity [88, 91, 92, 93, 94]. Genomic biomarkers should therefore be treated as substrate markers and interpreted through lineage, treatment history, and TIME state.

### 5.3 Functional DDR and Innate‐Sensing Biomarkers

Functional biomarker timing is critical. A RAD51 assay before treatment may identify HR restoration, whereas gamma‐H2AX or micronuclei shortly after therapy can document damage induction [195, 196]. IFN‐stimulated genes measured too early may miss sensing, whereas measured too late may reflect tolerance or systemic inflammation. Similarly, PD‐L1 induction can mean immune priming or adaptive resistance depending on whether CD8+ cells and antigen presentation increase in parallel. Biomarker protocols should therefore predefine sampling windows that match the expected kinetics of the selected drug and combination.

Functional markers show whether a tumor is actively processing DNA damage. RAD51 foci can assess HR function more directly than a static HRD scar. Gamma‐H2AX, phospho‐ATM, phospho‐CHK1, RPA loading, replication‐stress markers, and micronuclei can document target engagement and damage persistence [43, 67, 128, 197, 198, 199, 200]. These markers become more informative when paired with cytosolic DNA, cGAMP, STING/TBK1/IRF3 phosphorylation, IFN‐stimulated genes, MHC‐I induction, and ENPP1 or adenosine pathway activity [9, 20, 22, 33, 37, 128]. The key question is not whether treatment caused damage; it is whether damage moved along the immune‐conversion pathway.

### 5.4 Antigen‐Presentation, Immune‐Context, and Spatial Biomarkers

Spatial biomarkers can also guide therapeutic choice. A spatially inflamed tumor with high PD‐L1 and CD8‐tumor contact may be suited to checkpoint blockade after DDR priming. An excluded tumor with CD8 cells trapped in stromal margins may require radiotherapy, matrix remodeling, anti‐VEGF, or myeloid/stromal cotargeting. A tumor with HLA‐I LOH or B2M loss may not benefit from therapies that rely on classical CD8 recognition and may require NK‐cell, macrophage, bispecific, or CAR‐based strategies. Integrating spatial data can therefore prevent biologically implausible combinations.

Antigen presentation should be assessed as an independent layer. HLA Class I, B2M, TAP1/2, ERAP1/2, NLRC5, IFN–JAK signaling, and HLA‐I LOH can explain why high TMB or HRD does not translate into T‐cell killing [149, 150, 151, 152, 153, 167]. Immune‐context biomarkers such as CD8 density, Tregs, TAMs/MDSCs, NK cells, and PD‐L1 describe the cellular balance that therapy is attempting to reshape [96, 114, 120, 201]. B‐cell/TLS features and progenitor‐exhausted T‐cell states can provide additional information about immune response competence [124, 125, 126, 163].

Spatial biomarkers are especially important because inflamed and excluded tumors can look similar in bulk. Tumor‐CD8 contact, HLA‐CD8 geography, macrophage‐rich exclusion zones, fibroblast barriers, vascular access, and tertiary lymphoid structures can determine whether antigens are physically accessible to immune cells [90, 112, 113, 114, 163, 202]. Serial circulating markers such as ctDNA, cytokines, and IFN signatures are easier to collect but must be interpreted alongside tissue architecture when spatial access is central.

### 5.5 Composite and Modular Stratification Models

A modular model is also easier to validate than a large black‐box signature. Each module can be tied to a therapeutic action: repair dependency to DDR inhibitor choice; damage activity to dose; innate sensing to STING or ENPP1 strategies; antigen presentation to ICB or TCR/TIL suitability; immune geography to stromal/myeloid partners; and circulating dynamics to early stopping or escalation. The final clinical algorithm may be compact, but it should preserve this causal structure so that failure of one module can be recognized and corrected.

**FIGURE 5:**
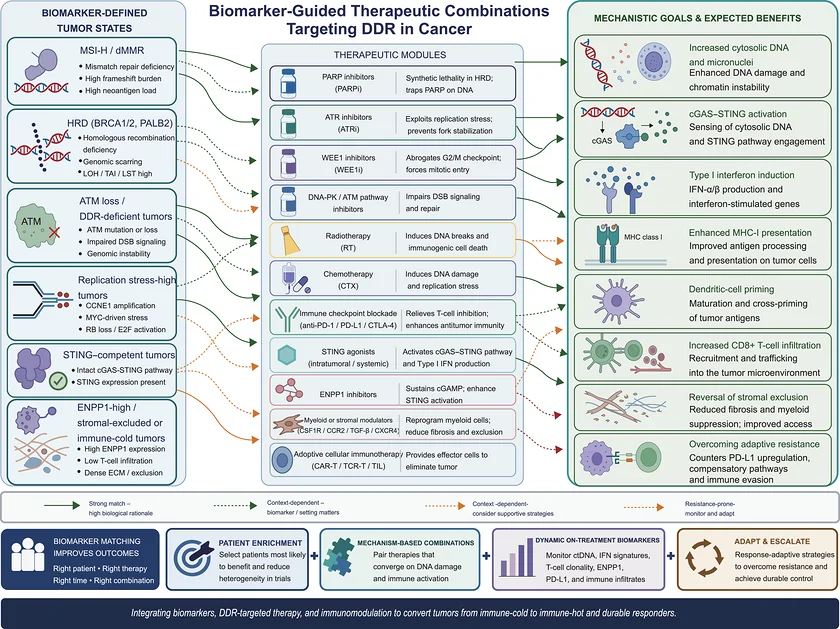
Biomarker‐guided therapeutic combinations targeting DDR in cancer. Biomarker‐defined tumor states, including MSI‐H/dMMR, HRD, ATM loss, replication stress‐high tumors, STING‐competent tumors, and ENPP1‐high or stromal‐excluded tumors, can be matched to rational therapeutic modules. Arrows indicate strength and context‐dependence of rationale, linking patient enrichment to mechanistic goals such as cGAS–STING activation, MHC‐I presentation, dendritic‐cell priming, CD8+ T‐cell infiltration, and reversal of stromal exclusion.

The most practical model is modular rather than monolithic. In some tumors, repair dependency dominates selection. In others, preserved innate sensing, antigen presentation, myeloid state, or spatial access is decisive. A baseline algorithm could begin with genomic DDR state and lineage, add functional repair and damage markers, assess innate‐sensing competence, determine antigen‐presentation integrity, and then classify immune geography as inflamed, excluded, or desert. On‐treatment sampling should then ask whether the selected therapy produced the intended transition. Table 5 summarizes candidate biomarker layers and evidence levels. Figure 5 integrates these biomarker layers with rational therapeutic modules and expected immunologic goals.

**TABLE 5:** Modular biomarker framework for DDR–immune stratification, with evidence levels and potential uses in adaptive trial design.

Biomarker layer	Representative readouts	What it answers	Evidence level	Use in trial design
Genomic DDR state [95, 102, 103, 158, 187, 188, 189, 190, 191]	MSI‐H/dMMR, BRCA1/2, PALB2, FA genes, ATM, CDK12, SETD2, HRD scars, TMB, and mutational signatures.	Is there a repair defect or mutational substrate that could create drug sensitivity or neoantigens?	Clinically established for MSI‐H ICB and selected PARPi uses; otherwise translational.	Baseline enrichment; stratify by tumor lineage and prior therapy.
Functional DDR activity [195, 196, 199, 200]	RAD51 foci, gamma‐H2AX, phospho‐ATM/CHK1, RPA loading, micronuclei, replication‐stress markers.	Is the tumor actively generating and processing damage after treatment?	Translational/preclinical, with selected clinical pharmacodynamic use.	Early go/no‐go decisions, dose and schedule optimization.
Innate sensing and IFN competence [20, 22, 33, 37, 128, 130]	Cytosolic DNA, cGAMP, STING/TBK1/IRF3 activation, IFN‐stimulated genes, ENPP1, and adenosine markers.	Can damage be converted into inflammatory signaling in tumor or host cells?	Translational; assay standardization incomplete.	Select DDRi–ICB, RT‐ICB, and STING agonist combinations; identify ENPP1‐high dampening.
Antigen presentation and escape [149, 150, 153, 160, 161, 162, 167]	HLA Class I, B2M, TAP1/2, ERAP1/2, NLRC5, IFN–JAK signaling, HLA‐I LOH, and immunopeptidomics.	Can newly generated antigens be processed and displayed?	Translational with growing clinical relevance.	Exclude or cotarget antigen‐presentation‐defective tumors; explain primary or acquired ICB resistance.
Immune contexture [90, 109, 120, 163, 201]	CD8 density, TPEX‐like cells, Tregs, TAM/MDSC, NK cells, TLS/B‐cell subsets, PD‐L1, and exhaustion markers.	Is the TIME effector‐rich, suppressive, excluded, or immune‐desert?	Clinically mature for selected markers; otherwise platform dependent.	Choose ICB, myeloid, stromal, or cellular therapy partners.
Spatial organization [112, 114, 164, 202]	Tumor‐CD8 contact, HLA‐CD8 geography, macrophage‐rich barriers, CAF/ECM exclusion, vascular access.	Can immune cells physically reach and kill malignant cells?	Hypothesis‐generating to translational.	Differentiate inflamed from excluded tumors; guide conversion strategies.
Dynamic/circulating monitoring [190, 198, 202]	Serial ctDNA, cytokine/IFN panels, immune‐cell phenotyping, on‐treatment biopsy, and spatial reassessment.	Is the treatment producing immune conversion or adaptive resistance?	Exploratory but increasingly feasible.	Adaptive randomization, early stopping, switch, or add‐on rules.

## 6 Future Perspectives: From Static Genotyping to Dynamic Immune‐State Monitoring

The unifying challenge for the field is to move from static genotyping to dynamic immune‐state monitoring. DDR genotypes are still essential, but they are only the starting state. Effective precision immuno‐oncology will require measuring how tumors, immune cells, and stromal compartments change after DDR‐directed therapy.

### 6.1 Dynamic State Transitions

State transitions may also differ between lesions in the same patient. A liver metastasis, lymph‐node lesion, and bone lesion can have different stromal density, myeloid composition, vascular access, and drug exposure. One lesion may become inflamed after DDR priming while another progresses through immune exclusion. Imaging, lesion‐specific biopsy and ctDNA kinetics can help identify whether resistance is systemic, lesion‐specific, or immune‐compartment‐specific. This is particularly important for radiotherapy and radionuclide strategies, where damage distribution is spatially patterned.

A tumor classified as HRD, MSI‐H, ATM‐deficient, or replication‐stress‐high before treatment may move into different immune states after therapy begins. One lesion may become inflamed and accessible; another may become PD‐L1‐high but T‐cell excluded; a third may suppress STING, degrade cGAMP or damage host lymphocytes. Capturing these state transitions requires baseline tissue, early on‐treatment tissue or blood, and progression samples when feasible [90, 110, 114, 198, 203, 204].

### 6.2 Adaptive Trial Design

Adaptive trials do not necessarily need complex multiomics for every patient. A feasible design could use broad sequencing and immunohistochemistry for baseline enrichment, then a focused on‐treatment panel measuring DNA damage, IFN signaling, HLA‐I/B2M, PD‐L1, CD8, and macrophage markers. More advanced spatial or single‐cell assays could be embedded in translational cohorts. This tiered approach would preserve biological insight while keeping the trial practical for multicenter enrollment.

Trial design should incorporate early pharmacodynamic decision points. A DDRi–ICB regimen could require evidence of target engagement, micronuclei, IFN signaling, MHC‐I induction, and preserved CD8 fitness before continuing. Patients who develop ENPP1‐high dampening, macrophage‐dominant suppression, or antigen‐presentation loss could be switched to STING agonist, myeloid, stromal, or cellular therapy strategies. Such designs would be more informative than broad all‐comer trials that test one static biomarker and one fixed schedule.

### 6.3 Feasible Implementation

For clinical adoption, assay harmonization will be as important as biological discovery. RAD51 foci, HRD scores, STING pathway markers, spatial CD8 proximity, and IFN signatures currently vary by platform, tissue quality, and threshold. Prospective trials should therefore define analytical cutoffs, pre‐analytical tissue handling, timing of on‐treatment biopsies, and minimum reporting standards. Without such standardization, promising DDR–immune biomarkers may remain difficult to compare across studies and difficult to use in routine practice.

**FIGURE 6:**
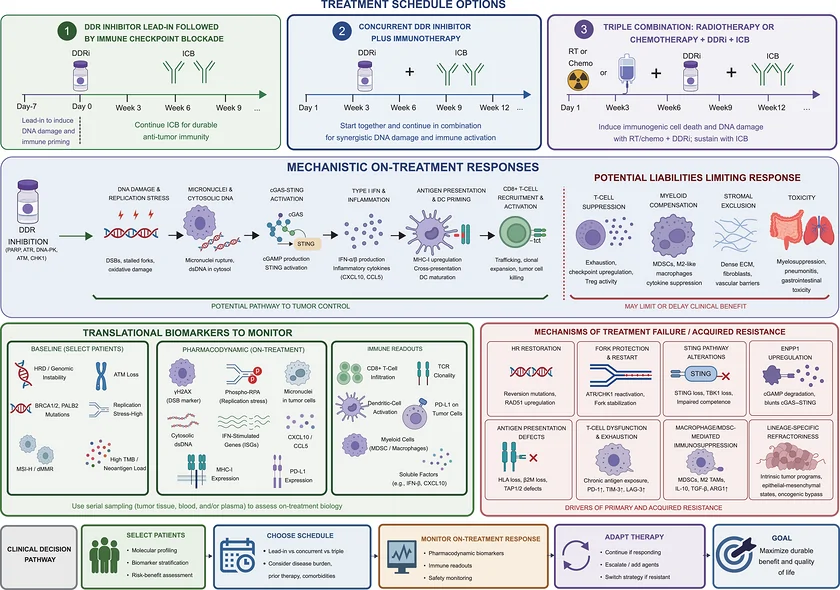
Clinical translation, treatment scheduling, and resistance monitoring for DDRi–immunotherapy combinations. The figure contrasts DDRi lead‐in, concurrent DDRi‐immunotherapy, and triple‐combination schedules, then links on‐treatment DNA damage, micronuclei, cGAS–STING activation, interferon, MHC‐I upregulation, dendritic‐cell priming, and CD8+ T‐cell activation with biomarkers and resistance mechanisms. The clinical decision pathway emphasizes patient selection, schedule selection, serial monitoring, and response‐adaptive therapy.

Implementation will require simplifying multiomics into deployable assays. Routine sequencing can identify MSI, HRD, TMB, and repair genes. Immunohistochemistry or multiplex immunofluorescence can assess HLA‐I, B2M, CD8, PD‐L1, macrophages, and spatial access. Focused RNA panels can measure IFN, chemokine, and ERV signatures. ctDNA and blood immune profiling can track tumor burden and systemic immune effects. The best future biomarkers will not be the largest signatures, but the smallest combinations that reliably identify a modifiable DDR–immune state. Figure 6 summarizes a clinically oriented workflow that connects treatment scheduling, pharmacodynamic monitoring, resistance detection, and response‐adaptive therapy.

## 7 Conclusions

Targeting the DDR in cancer now means more than exploiting synthetic lethality. DDR defects and DDR inhibitors can kill tumor cells, generate cytosolic nucleic acids, activate innate sensing, increase antigen presentation, and cooperate with immunotherapy. They can also induce chronic STING tolerance, checkpoint adaptation, lymphocyte stress, myeloid suppression, stromal exclusion, and resistance. The central translational problem is therefore damage‐to‐immunity conversion: determining when a DNA damage signal becomes a useful immune event and when it becomes suppressive noise.

The next stage of DDR‐directed therapy should combine target‐specific drug selection with tumor lineage, pharmacologic scheduling, host immune‐cell protection, and integrated biomarkers. Genomic DDR lesions identify potential vulnerabilities; functional and spatial biomarkers determine whether those vulnerabilities are immunologically exploitable; dynamic monitoring shows whether therapy has moved the tumor toward productive immune engagement. This framework may help transform DDR targeting from a static genotype‐driven approach into a state‐guided strategy for precision cancer therapy.

## Author Contributions

**Yihuan Qiao**: conceptualization, investigation, data curation, and writing – original draft. **Boyu Kang**: investigation, data curation, and writing – original draft. **Jian Zhang**: conceptualization, supervision, and writing – review and editing. **Rui Zhang**: supervision and writing – review and editing. **Jipeng Li**: conceptualization, supervision, project administration, and writing – review and editing. All authors read and approved the final manuscript.

## Funding

This work was supported by the National Clinical Research Center for Digestive Diseases (2026NCRCDDZZ08) and the Shaanxi Special Support Plan Scientific and Technological Innovation Leader (4457475042BR2200OY).

## Ethics Statement

This review did not involve new studies with human participants or animals performed by the authors.

## Conflicts of Interest

The authors declare no conflicts of interest.

## Data Availability

Data sharing is not applicable to this review article because no new datasets were generated or analyzed.
